# Conceptualizing the Role of Target-Specific Environmental Transformational Leadership between Corporate Social Responsibility and Pro-Environmental Behaviors of Hospital Employees

**DOI:** 10.3390/ijerph19063565

**Published:** 2022-03-17

**Authors:** Yuwei Deng, Jacob Cherian, Naveed Ahmad, Miklas Scholz, Sarminah Samad

**Affiliations:** 1 School of Marxism, Heilongjiang University, Harbin 150080, China; dengyuwei@dqnu.edu.cn; 2 School of Mechatronics Engineering, Daqing Normal University, Daqing 163111, China; 3 College of Business, Abu Dhabi University, Abu Dhabi P.O. Box 59911, United Arab Emirates; jacob.cherian@adu.ac.ae; 4 Faculty of Management Studies, University of Central Punjab, Lahore 54000, Pakistan; naveeddgk2010@gmail.com; 5 Faculty of Management, Virtual University of Pakistan, Lahore 54000, Pakistan; 6 Division of Water Resources Engineering, Department of Building and Environmental Technology, Faculty of Engineering, Lund University, P.O. Box 118, 221 00 Lund, Sweden; 7 Department of Civil Engineering Science, School of Civil Engineering and the Built Environment, University of Johannesburg, Kingsway Campus, Aukland Park, P.O. Box 524, Johannesburg 2006, South Africa; 8 Department of Town Planning, Engineering Networks and Systems, South Ural State University, 76, Lenin Prospekt, 454080 Chelyabinsk, Russia; 9 Institute of Environmental Engineering, Wroclaw University of Environmental and Life Sciences, ul. Norwida 25, 50-375 Wroclaw, Poland; 10 Department of Business Administration, College of Business and Administration, Princess Nourah Bint Abdulrahman University, Riyadh 11671, Saudi Arabia; sarminasamad@gmail.com

**Keywords:** altruistic values, decarbonization of healthcare, employees and organizational success

## Abstract

The healthcare sector throughout the world is identified for its outsized carbon footprint. Despite the mounting importance of employees’ pro-environmental behavior (PEB) for decarbonization, the role of PEB in a healthcare context was less emphasized previously, especially in a developing country context. To address this knowledge gap, the current work was carried out to examine the relationship between a hospital’s corporate social responsibility (CSR) initiatives and PEB with the mediating effect of environmental-specific transformational leadership (ESTL). At the same time, the conditional indirect effect of altruistic values (AV) was also considered in the above relationship. The data were collected through a questionnaire by employing a paper-pencil method from the hospital employees (*n* = 293). By considering the structural equation modeling, the hypothesized relationships were validated. The results indicated that CSR directly (*β*1 = 0.411) and indirectly, via ESTL, (*β*4 = 0.194) influenced the PEB of employees. It was also realized that A.V produced a conditional indirect effect in this relationship (*β*5 = 0.268). This work tends to help a hospital to improve its environmental footprint through CSR and ESTL. Moreover, the current work also highlights the role of employees’ values (e.g., A.V) to guide the environment-specific behavior of employees.

## 1. Introduction

Concern for the ecological environment has become a contemporary topic of academic debate. At the same time, the increasing climate change issues have led organizations to pay attention to their environmental strategies [1]. Given that the role of businesses is critical to improving a country’s ecological footprint, the recent organizational management literature has received mounting scholarly attention to highlight different factors that can help an organization to improve its carbon footprint [2,3].

In this vein, recently, the role of employees for the effective management of an organization has been discussed at many levels. From an environmental perspective, it was argued that for effective environmental strategy implementation, employees’ perceptions of environmental problems [4,5] and their responsible behavior [6] are of seminal importance. Undoubtedly, employees are central to an organization in terms of achieving its green initiatives. Indeed, organizational effectiveness for sustainability-related policies is highly dependent on employees’ behavior, especially their discretionary behavior (extra-role behavior), which does not fall under formal job obligation systems, for instance, formal reward and performance evaluation [7], yet such behaviors are important for the success of an organization.

Research shows that the eco-friendly behavior of employees can enhance the sustainable performance of an organization. Generally, literature regards environment-specific behavior, eco-friendly behavior, and sustainable behavior as pro-environmental behavior (PEB). Kollmuss and Agyeman [8], defined PEB as an individual’s adaptive behavior to avoid actions that can harm nature and the built environment. Prior research shows that PEB as an adaptive climate change behavior can be helpful in dealing with the environmental issues effectively. Despite the mounting importance of PEB in academic literature, it seems that a consensus has not yet been reached to determine what drives the PEB of employees. Nevertheless, in broad terms, the literature has stated that different organizational and personal factors contribute to the formation of individual behavior [9]. Therefore, to explain the PEB of employees in an organizational context, both organizational and personal factors should be considered.

In this regard, the role of an organization’s corporate social responsibility (CSR) engagement as an organizational factor to influence employees’ behavior has been discussed previously [10]. Specifically, it was argued that CSR can drive the extra-role behavior of employees. For example, a positive link between CSR and employees’ organizational citizenship behavior (an extra role behavior) was well discussed in prior literature [11,12]. However, approaching CSR to explain employees’ PEB is something that became a topic of academic debate just recently. Moreover, such a relationship in the context of Pakistan (a developing country) which has been a bad victim of environmental issues [13], remained an understudied research area. Thus one specific aim of this work is to explain employees’ PEB from the standpoint of CSR in Pakistan.

Another organizational factor that has been well discussed in the literature to influence employees’ behavior is the leadership style in an organization [14,15]. Corporate leaders not only influence the formal behavior of employees but also produce a significant impact on employees’ extra-role behavior, including PEB [16,17]. Among several leadership styles, transformational leadership received greater scholarly attention in organizational management literature from the employees’ perspective [18,19]. Nevertheless, a shift in the field of transformational leadership was recently noted. That is, shifting from a general approach toward transformational leadership to a target-specific leadership style. In this vein, Barling, et al. [20] were among the first who recognized the target-specific role of transformational leadership from the standpoint of occupational safety. Since then, various subsequent explanations of target-specific transformational leadership have been provided [21,22]. However, such a target-specific transformational leadership approach was not well applied to explain employees’ PEB. Therefore, following this target-specific research paradigm, the present study also aims to test the mediating role of environmentally specific transformational leadership (ESTL) to influence PEB.

At a personal level, the literature also notes the role of values to form individual behavior [23]. From an environmental perspective, the role of altruistic values (A.V) was also highlighted [24]. Indeed, A.V was conceptualized as a personal value of an individual value structure that urges a person to contribute to the wellbeing of others including society and the biosphere [25]. Griskevicius, et al. [26] indicated that, as with most environmental behaviors, PEB has an intrinsic characteristic of altruism. Hartmann, et al. [27] argued that A.V plays a significant role in guiding the PEB of individuals.

However, as the values only provide a general guideline to behavior formation, in investigating the direct link between A.V and PEB it is worthwhile to note their moderating role in a specific relationship [28]. Therefore, this study proposes A.V as a moderator to produce a conditional indirect effect between the mediated relationship of CSR and PEB.

To test the above-hypothesized relations, this study selected the healthcare sector, which is known for its outsized carbon emissions. Given that the environmental issues have already been worsening in several regions of the world, the healthcare sector needs to significantly improve its environmental footprint [29]. Reflecting this to the context of Pakistan, a country where environmental issues are becoming more intense with each passing year, the country’s healthcare sector needs to take different initiatives to mitigate its harmful impact on the environment. Hospitals in Pakistan generate huge waste on daily basis, including plastic, textile, food, glass, and others [30]. Given that the hospital industry in Pakistan employs a huge workforce, it is worthwhile to mitigate the negative environmental effect of this segment by promoting the PEB among employees through CSR and ESTL.

The current work fills the knowledge gaps in the following ways. First, this work advances the field of organizational management and sustainability by investigating the target-specific transformational leadership approach by proposing the mediating effect of ESTL between CSR and PEB. In this vein, as already stated, most of the prior literature considered transformational leadership as a general approach to influence employees’ behavior. Though some studies investigated a target-specific impact of transformational leadership from an environmental perspective [16,31], such studies are sparse to reach a consensus. Specifically, the role of ESTL in the healthcare segment in the CSR framework was not realized previously. Second, to the best of the authors’ knowledge, this work is the first one investigating the role of CSR, ESTL, and A.V to spur PEB in the healthcare sector of Pakistan, which was not tested earlier. Third, most PEB studies in healthcare sectors from CSR leadership perspectives were conducted in developed or advanced countries previously [32,33,34]. However, the healthcare segment from most of the developing countries remained an under-explored terrain. Last, most of the prior literature under environment management focused on the manufacturing sector [35,36]. These studies were logical because manufacturing industries produce a larger environmental impact through their industrial operations. However, neglecting the services sector including a healthcare system is unwise, as current climatic conditions throughout the globe require measures on every ground.

**Figure 1:**
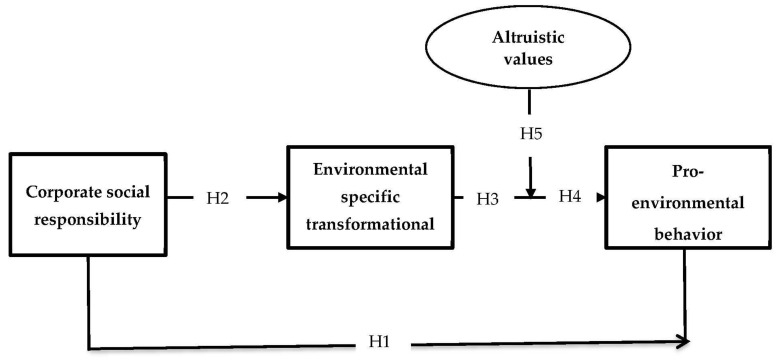
The hypothetical model with arrowhead indicating different relationships and proposed hypotheses.

This work is grounded in the theory of social learning proposed by Bandura and McClelland [37]. This theory argues that individuals’ social behavior is influenced by observing and copying others’ behaviors. Leadership studies put significant emphasis on this theory with regard to influencing the behavior of employees. In this vein, it was found that leaders in a workplace context shape the behavior of followers (the employees) through a social learning process [16]. Reflecting the process of social learning into the current work’s theme, when employees observe the conduct of their leader to preserve the environment through different acts of resource conservations, they learn such discretionary behaviors on their part through a social learning process as a result of observation. This theory also states that individuals change their behavior in response to their surroundings. From this aspect, the CSR engagement of a socially responsible organization creates a “caring for others” work environment. When employees observe this caring orientation of their organization, their own behavior is influenced, and thus they show the same “caring for others” intentions. Hence, they are expected to act pro-socially and to utilize organizational resources in a manner in which wastage or unnecessary usage is avoided to preserve them for future generations (caring for others). The hypothetical framework of the current work is provided in Figure 1. 

## 2. Material and Methods

### 2.1. Hypotheses

Earlier studies have documented that CSR as an organizational factor can be an enabler for employees’ PEB [38,39]. The work of Vlachos*,* et al. [40] has asserted the notion that “employees respond positively to CSR” to conclude that employees are engaged in different extra-role behaviors in an organization due to its ethical commitment. Specifically, employees’ observation of their organization towards CSR activities was positively linked to enhance their engagement and to fully embrace the CSR orientation of their organization at their level too. Connecting this to the current work, it may be argued that employees’ CSR perceptions can increase their motivation level to support their organization by performing different extra-role activities. As PEB falls under the category of extra-roles, it is expected that the social commitment of an organization can drive employees’ PEB. This view is also supported by previous CSR scholars [41,42]. Furthermore, referring to the theory of social learning, it can be argued that the employees in a socially responsible organization observe the social orientation of their organization. This observation then serves as a guide for their behavior formation. Specifically, when they observe that their organization is showing greater commitment in the larger interest of all stakeholders, they learn this socially responsible behavior through the social learning process [43], which eventually leads them to act pro-environmentally [44]. A ‘caring for others’ environment, as a result of CSR, motivates employees to practice the same on their part [45]. Moreover, referring to Dewhurst*,* et al. [46], the concept of CSR is more suitable to be positively linked with the extra-role behavior of employees. In this regard, as the CSR commitment of an organization is perceived by employees as an extra-role commitment to benefit society and the environment, the social learning process of employees helps them to imitate this extra-role commitment at their level as well. Thus, it is expected that in response to the CSR engagement of their organization, employees also become socially responsible and show better engagement to act pro-environmentally. Therefore, the following hypothesis may be stated:

**Hypothesis** **1** **(H1).** *Employees’ CSR perceptions for their socially responsible organization can positively drive PEB.*

The literature highlights the importance of an effective leadership style from an organizational perspective. Socially responsible organizations treat their leaders as valued resources and attempt to convert them into happy leaders who willfully put forth the organizational objectives, including the sustainability objectives [47,48]. A positive link between CSR and leadership style has also been reported in previous works [49,50]. In this regard, transformational leaders are at the heart of a socially responsible organization to implement different social initiatives and to clarify the followers about the seriousness of an organization for CSR-related objectives [51]. Following the theme of the current work, the CSR orientation of an organization, especially for the environment, is expected to influence ESTL positively through a social learning process. Though literature documents the benefits of a transformational leader for organizational effectiveness [52], it is expected that while working in a socially responsible organization that shows a greater commitment to the environment, the environmental preference of a transformational leader may be further enriched. Therefore, the current study states the following hypothesis.

**Hypothesis** **2** **(H2).** *There is a direct association between the CSR orientation of an organization and ESTL.*

Corporate leaders can significantly influence various traditional corporate outcomes, including employees’ behaviors, corporate tasks, financial objectives, and safety performance [20,53]. At the same time, leaders also influence some emerging outcomes on the part of employees’ behavior, including their environment-friendly behavior. It was realized in prior literature that leadership styles that a leader exhibits towards the environment can motivate employees’ PEB [16,54]. Perhaps, among different leadership styles in the context of business, transformational leadership has received mounting scholarly attention to managing an organization effectively by influencing employees’ attitudes and behaviors [18,19]. As compared to different traditional styles of leadership, including transactional leadership, a transformational leader is one who is more capable of inspiring and motivating his followers by depicting the vision of an organization, showing a caring attitude for the followers, and influencing their behavior by setting himself as a role model [55]. In line with a target-specific research paradigm in the field of transformational leadership, some recent studies have established a positive link between ESTL and employees’ PEB [17,56]. Specifically, ESTL urges employees to endorse PEB in a workplace environment through the process of social learning. In this vein, the employees observe and learn that their leader prioritizes environmental issues to improve the environmental footprint of their organization [31]. Environmental researchers have stressed that the process of social learning can explain the exchange of relationships between a leader and followers [16,57]. To explain further, it was mentioned that a transformational leader with an environment-specific approach shows a higher inclination towards the environment when encountered by different operational demands, for instance, environment versus efficiency. In such situations, a transformational leader shows that environmental issues are more important over efficiency concerns [58,59]. In return, by observing the conduct of their leader as a role model and through the process of social learning, employees learn that environmental issues are prioritized, which ultimately help them to form their environment-specific behavior.

When applied to the current work context, a socially responsible organization shows a higher concern to preserve the environment for future generations. Under this CSR orientation, a transformational leader with a high environmental orientation effectively communicates and implements this social concern of his organization to employees. Employees, on the other hand, learn this organizational philosophy not only by observing the environmental values of their socially responsible organization, but through the presence of an ESTL that provides them with a further explanation to guide their responsible behavior. Hence the following hypotheses may be stated.

**Hypothesis** **3** **(H3).** *Environmental specific transformational leadership style can be positively linked with employees’ PEB.*

**Hypothesis** **4** **(H4).** *The link between CSR and employees’ PEB is mediated by environmental specific transformational leadership.*

Although the mainstream literature recognizes the role of altruism for behavior formation in a non-organizational context, the current study aligns with the argument of Elshuis [60], who posited that altruism can be helpful to influence employees’ behavior in an organizational context. Considering values are culturally shared, a socially responsible organization gives a central place to environmental values, and hence develops an organizational culture that shares environmental values with the employees [61]. Employees, on the other hand, also consider organizations with values that are in congruence with their personal values. Though values guide behavior at a personal level, values are also influenced in the milieu of interaction in an organization. In this this regard, the A.V of employees is centrally placed to strengthen the pro-environmental orientation of an employee.

From an environmental perspective, values, specifically A.V, focus on the collective wellbeing of others (i.e., considering the benefit of society and the biosphere) [62]. Referring to the work of Stern and Dietz [63], this study argues that the environmental values of employees can motivate them to act pro-environmentally in a workplace. Moreover, values are assumed to be stable in nature, indicating the reason why several scholars stress the importance of values in shaping individual behavior. Indeed, it is also assumed that values are viewed similarly universally [64], and can predict the same individual behavior patterns in different cultures. Although the importance of values in influencing individual behavior has been significantly noted by different scholars in the available literature, however, values only provide a general foundation to develop a specific behavior. This is why different researchers have stressed their indirect potential for behavior formation rather than stressing their direct potential. Hence, the moderating role of altruistic values has received considerable attention from different scholars [61,65]. In the current settings, the CSR commitment of a firm inculcates the feelings of ‘care for others’ in employees, which in turn urges them to act pro-environmentally. When the role of A.V is also considered, it is expected that such a relationship get strengthened to a further level. Therefore,

**Hypothesis** **5** **(H5).** *The presence of altruistic values moderate the mediated relationship between CSR, environmentally-specific transformational leadership, and PEB, such that the relationship is stronger in the presence of altruistic values.*

### 2.2. Participants and Procedure

This study targeted Lahore city to collect the data from different hospitals. This city was considered representative due for two reasons. First, Lahore is globally known for its poor air quality, as the city was ranked as the highest several times in the list of most polluted cities globally [66]. Characterized by a population in the multi-millions, the public health of the masses in this city is at risk due to environmental hazards. Second, Lahore is a city where a vast number of public and private hospitals are located, which makes it logical to select this city as a representative one from the context of the current research.

A self-administered questionnaire (paper-pencil technique) was employed as a data-collecting instrument. Before finalizing such an instrument, the questionnaire items were assessed by experts in the field. Only after receiving valuable feedback from the experts was the final version of the questionnaire provided to the survey respondents [67,68]. The questionnaire was divided into two main parts. The first part was associated with the general demographic, and the second part included the main survey items. The authors also observed the ethical guidelines of the Helsinki Declaration as proposed by different prior researchers [69,70]. Initially, 500 questionnaires were provided to different respondents, and 293 were eventually collected by the authors. Thus the true response rate remained at 58.6%.

The data were collected in three waves. This was done to overcome the issue of common method bias (CMB). In the first wave, the employees were asked to provide their demographic information (age, gender, education, etc.), and the A.V-related information. In the second wave, the employees were asked to rate their perceptions of their leader and the CSR engagement of their hospital. Lastly, the employees with managerial ranks were asked to rate their PEB perceptions of an employee. These steps to collect the data are in line with the recommendations of Qing*,* et al. [71]. The data collection activity was carried out from August to November 2021. In terms of demographic information, male respondents constituted 58.39%, whereas 89% of employees were between the ages of 22–40 years. The employees with leadership positions were 39% in this survey. Most of the employees (66%) have work experience of between one and five years.

### 2.3. Measures

There were four constructs in this study for which the authors employed the already existing scales. For example, a 12-item CSR scale was adapted from Turker [72]. A recent study by Ahmad, et al. [73] also used this scale to measure employees’ CSR perceptions. The alpha value (α) of this construct was 0.940. One item from this scale was “Our hospital makes investments to create a better life for future generations." A 12-item scale to measure a leader’s PEB perception of an employee was taken from Lamm, et al. [74] with an α value of 0.942. A sample item includes “He/she is a person who uses scrap paper for notes instead of fresh paper”. Likewise, the scale of A.V was adapted from De Groot and Steg [75], which included eight items. This scale was also employed by different extant researchers. For example, Lee, Kim, Kim and Choi [62] used this scale to measure the A.V of individuals. In the hospitality context of Pakistan, a recent study by Shao, Mahmood and Han [28] also employed this the same. For this scale, the employees were asked to rate their perception ratings with regard to importance. One item from this scale was “as a guiding principle in my life, I consider pollution prevention.” An α value of 0.914 was obtained for this scale. Specifically, this adapted scale ranged from 1 to 7 where 1= not important, and 7 = extremely important.

Lastly, the scale of ESTL was borrowed from [76]. This scale included a total of 12 items to measure employees’ perceptions of their leader. Among these 12 items, three items were related to employees’ perceptions of their leader in terms of idealized environmental influence, and three items were concerned with employees’ perceptions of their leader to influence environmental inspirational motivation. Likewise, three items were related to the extent to which employees perceive their leader as a source of environmental intellectual stimulation. Lastly, three items were related to the individualized environmental consideration of a leader in a workplace. This scale showed an α value of 0.929, which was significant. One sample item was “My leader acts as an environmental role model.” All responses were recorded on a 7 point Likert scale. Lastly, to assess the non-response bias, the authors compared the demographic information of the respondents with full information with the respondents with partial information and observed no significant difference between the two. Hence, no specific non-response bias was found.

## 3. Results

### 3.1. Construct Evaluation

The authors conducted a common latent factor (CLF) test to see if a CLF can explain a sheer amount of total variance (beyond 50%). In this vein, the results showed that no such CLF existed, implying that a CMB issue was not critical in this survey [28]. Moreover, the authors also noted the standardized regression weights between the two models, a model with no CLF (base-line), and a model with a CLF. It was noted that no significant difference (>0.2) in the regression weights of each factor between the two models was observed.

Prior to hypotheses testing, the authors, first of all, evaluated each construct for validity and reliability. In this process, the standardized factor loadings (λ) of all four constructs of this study were observed against the standard criterion of 0.5 and ideally 0.7. These results of factor loadings can be seen in Table 1. As per the stated results, most of factor loadings were beyond 0.7 [77]; nevertheless, one factor loading from ESTL and one from PEB were below 0.5 (ESTL 08, λ = 0.47; PEB 04, λ = 0.39). Therefore, these items were removed from the analysis, and all stages of data analysis were performed with total items of 11 for ESTL and 11 for PEB. After verifying that no issue exists in factor loading for each item, the authors were able to calculate convergent validity based on these factor loadings. To do this, average-variance-extracted (AVE) was calculated in each case, which was then compared against the standardized values of 0.5. It was noted that the AVE for each construct were beyond 0.5 (AVE for CSR = 0.580, PEB = 0.649, A.V = 0.634, and ESTL = 0.582). These values of AVEs were enough to statistically establish that convergent validity for all four constructs was significant. Likewise, the standardized factor loadings were also helpful to compute the composite reliability (C.R) for the constructs of this study. These values have also been presented in Table 1. It can be seen that the C.R values in every case were above 0.7 (C.R for CSR = 0.943, PEB = 0.953, A.V = 0.933, and ESTL = 0.939), which were significant.

### 3.2. Correlations and Divergent Validity

After verifying the significance of factor loadings, convergent validity and C.R, the authors next performed a correlation analysis to see correlation nature and magnitude among different pairs. Table 2 shows the output of correlation analysis for different cases. For example the correlation value (*r*) between CSR and PEB (*r* = 0.398, *p <* 0.01), between CSR and A.B (*r* = 0.377, *p <* 0.01), between CSR and ESTL (*r* = 0.396, *p <* 0.01) were all positive and significant. Moreover, to verify the divergent validity of each construct, the square root of AVE (sqAVE) for a construct was compared with the correlation values. In this vein, if correlation values were found lesser than the sqAVE value, then the divergent validity is established. To explain further, the sqAVE value of CSR was 0.762 which was superior to the correlation values (0.398, 0.377, and 0.396). Hence, it was established that the items of one construct were not the same as in other cases, or put simply, they were dissimilar. Lastly, different measurement models were developed in AMOS compared with the hypothesized model (4-factor). It was revealed that the hypothesized model was the most significant compared to the alternate models. These results are presented in Table 3. It can be seen from the results that the model fit indices were more suitable for the hypothesized model compared to alternate models. For example, a *χ*^2^/*df* value less than 3 is considered significant, however, a smaller value less than 2 is desirable. In this respect, for the hypothesized model, *χ*^2^/*df* = 1.903 showed a significant value. Similarly, for NFI and CFI, a value greater than 0.9 is considered significant. In this respect NFI = 0.957 and CFI = 0.959, which were superior compared to alternate models. Likewise, the RMSEA value should be less than 0.08, however, a value less than 0.05 is desirable. In this vein, the hypothesized model produced an RMSEA = 0.039, which was significant.

### 3.3. Hypotheses Validation

Lastly, the hypothesized framework of this study was tested for acceptance or rejection by employing structural equation modeling (SEM) in AMOS. SEM is a second-generation data analysis tool to analyze complex models (like in the current study). The advanced features of SEM and flexibility options make its candidature more attractive. This is why SEM is a preferred choice of contemporary data scientists [78,79,80,81]. In this regard, the structural model was developed three times in AMOS. Firstly, a direct effect model was developed to see the direct relationships to validate H1, H2, and H3. In this model, ESTL was not identified as a mediator, rather ESTL and CSR were treated as independent constructs to influence PEB. The level of significance was set to 95%. In this vein, and the statement of H1 was “employees CSR perceptions for their socially responsible organization can positively drive PEB”. To validate this hypothesis, the authors considered the results of Table 2 (*r* = 0.398; *p* < 0.01) and Table 4 (beta value—*β*1 = 0.411; *t* = 10.538; *p* < 0.01). These outcomes provided statistical support to accept the statement of H1. Furthermore, the same above process was repeated to validate H2, and it was found that H2 was also significant (*r* = 0.398; *β*2 = 0.407; *t* = 10.710; *p* < 0.01). Thus the statement of H2 that “there is a direct association between the CSR orientation of an organization and ESTL” was accepted. Lastly, the statistical results of H3 were also significant (*r* = 0.482; *β*3 = 0.476; *t* = 14.000; *p* < 0.01) and hence it was verified that ESTL significantly predicts PEB.

After evaluating the direct effect model and validating H1, H2, and H3, the author developed the structural model again in the second phase. This time ESTL was identified as a mediator in the model, whereas CSR remained an independent construct. In this respect, the bootstrapping option in AMOS was employed by considering a larger bootstrapping sample of 2000, as recommended by different researchers [82,83,84]. Moreover, a biased corrected 95% confidence interval was also considered during this stage of the structural model. Table 5 shows the results of the mediated structural model. As per the results, it was realized that ESTL partially mediates between CSR and PEB (CSR→ESTL→PEB: *β*4 *=* 0.194, *p* < 0.01). Moreover, the mediation effect explained the nearly 47% variance in PEB. These results statistically established that ESTL is a significant mediator between CSR and PEB. Hence, according to the statement of H4, it was verified that ESTL mediates between CSR and PEB. Thirdly, the same previous structural model was considered again to evaluate the conditional indirect effect of A.V in the above-mediated relationship. This time, A.V was included in the model as a moderator between the mediated relationship of CSR and PEB via ESTL. The improvement in the beta value (*β*5 = 0.268) indicates that the relationship was strengthened in the presence of A.V, implying that A.V produces a significant conditional indirect effect in the mediated relationship between CSR and PEB via ESTL. Therefore, the statement of H5 was also verified.

## 4. Discussion

It was found that CSR can significantly drive employees’ PEB (*β*1 = 0.411) in a hospital context. Early research in the same domain also provides support to this result of the current work [45,84,85]; nevertheless, the current context (a healthcare context) was less emphasized in the prior literature. Given that the hospitals throughout the globe are identified as a source of greenhouse gas emissions, it was important to carry out this work. Moreover, the current work extends the debate by arguing that a transformational leader with environmental concerns shows a high preference to preserve the environment and conveys to their followers that environmental issues are preferred in a socially responsible hospital. When employees observe that their transformational leader shows a greater concern to preserve the environment through his actions and conduct, they learn this orientation of their leader, which ultimately guides them to act pro-environmentally. The early work of Robertson and Carleton [16] also highlighted the effectiveness of ESTL to drive employees’ PEB; however, the healthcare context was not tested previously. To this aspect, the results of this work showed that ESTL not only influences employees’ PEB (*β*3 = 0.476) directly, but that it also produces a mediating effect (47%) between CSR and employees’ PEB. This work also discusses the critical role of values, especially the A.V of employees, in fostering their PEB. In this vein, though the early work documents a positive link between individual values and behaviors [86], even a positive link between values and PEB was also reported earlier [87]; however, the role of A.V in a healthcare context was missed earlier. To this aspect, the findings of this work showed that A.V significantly influences the above-mediated relationship as a moderator (*β*5 = 0.268).

The current work tends to advance the literature on organizational and environmental management from a CSR and leadership perspective. In this regard, this work offers different critical theoretical implications. First, the current work is one of the sparse works that follow a target-specific transformational leadership approach from the standpoint of the environment that previously remained an unattended terrain. Second, this work adds to the prior literature on CSR, environmental management, and leadership from a healthcare perspective that was not previously focused on. Third, another important theoretical advancement of this work lies in its approach to individual values, especially A.V, in terms of fostering employees’ PEB in a CSR framework. The early research shows the seminal role of A.V to spur employees’ PEB; however, the conditional indirect role of A.V in a CSR framework was not discussed. Lastly, this work extends the debate on businesses’ concern for the environment in the context of a developing country, whereas most of the prior work was conducted in developed countries.

The hypothesized framework of the current analysis offers some important practical implications for the healthcare sector of Pakistan. First, as concern for the environment has been mounting at all levels, the healthcare sector of Pakistan can effectively address its environmental issues by the employees to act pro-socially in response to CSR and ESTL. In this aspect, the role of leadership is critical for a hospital to implement its sustainability initiatives at different levels. Realizing the trainability of transformational leadership, the management of a socially responsible hospital is suggested to incorporate greening initiatives into its leadership development programs and training courses. This will undoubtedly help the hospital leadership to improve their capability in solving environmental issues by acting as role models to the followers in terms of acting pro-socially.

Similarly, another aspect of leadership pertinent to the current work’s theme is to integrate the environmental values into the self-construction of an employee’s work by explaining the seriousness of environmental issues so that employees can raise their environmental concern by strengthening their AV, which then provides additional motivation to employees to act pro-environmentally. In this regard, the role of leadership is very important to activate employees’ environmental motivation by communicating to them that their socially responsible hospital prioritizes environmental interest. Thus, employees, being the important members of such a hospital, are encouraged to support their hospital’s sustainability initiatives.

The findings of this work verify the important role of A.V in influencing employees’ PEB in a CSR framework. This finding has an important practical implication for a hospital. That is, a hospital needs to revisit its recruitment and selection procedures by attaching greater importance to environmental values in the assessment process of an individual. This assessment will be helpful for a hospital to promote a culture of eco-friendly behavior among employees. This is because when employees join a socially responsible organization with a high environmental orientation, their social learning process guides them to practice the same on their part. To this end, employees with high environmental value (A.V) will show a greater commitment to supporting the sustainability initiatives of an organization by adopting sustainable work practices.

### Limitations

Despite the fact that this work contributes at a theoretical and practical level, it still faces some limitations. At the same time, these limitations may motivate future researchers to extend the current debate in the future. Specifically, the following are some limitations of the current work. First, the current work selected only one city in Pakistan to collect the data. Though the selection of Lahore city was logical to serve the current research purpose, geographic concentration may still be considered a limitation in the context of generalizability. In this regard, the authors suggest that future researchers add more cities (like Karachi, Faisalabad, and others) to claim better generalizability of the results. Second, the data of the current survey was cross-sectional, limiting the causality of the relationships (though all relationships were significant). In this vein, it is suggested to opt for a longitudinal data design in future studies to address the limitation of cross-sectional data design. Third, the study employed perceptual measures of CSR; though a plethora of studies employed this approach, the authors still feel that employing the actual measures of CSR may generate more accurate results.

## 5. Conclusions

The current work helps the hospital sector of Pakistan to improve its carbon footprint through employees’ PEB as a result of CSR and ESTL. Given that the environmental quality of Pakistan has been declining each year, and considering the outsized carbon footprint of the healthcare sector, the results of the current draft provide a way forward to deal with climate change issues through CSR and ESTL. Furthermore, the results of the current work also highlight the important role of a target-specific transformational leadership approach with the specific consideration of environmental management. To this end, the effectiveness of ESTL to deal with environmental issues was verified. Thus, the management of hospitals needs to give rise to the environmental values of their leaders through different training and seminars. The hospitals need to realize that CSR orientation by itself is not enough to improve the environmental situation, as the leadership has a seminal role to play in the effective implementation of a CSR strategy by aligning the workforce with an organization’s vision. Lastly, the hospitals need to realize the potential role of individual values, especially with regard to employees, because employees with high altruism are more committed to act pro-environmentally in an organization. 

## Figures and Tables

**Table 1:** Output of construct evaluation.

	λ	λ^2^	S.E	T. Values	E-Variance	AVE	C.R
CSR						0.580	0.943
	0.716	0.513	0.053	13.51	0.487		
	0.724	0.524	0.051	14.20	0.476		
	0.767	0.588	0.048	15.98	0.412		
	0.748	0.560	0.047	15.91	0.440		
	0.762	0.581	0.049	15.55	0.419		
	0.755	0.570	0.047	16.06	0.430		
	0.783	0.613	0.052	15.06	0.387		
	0.792	0.627	0.042	18.86	0.373		
	0.816	0.666	0.040	20.40	0.334		
	0.826	0.682	0.038	21.74	0.318		
	0.719	0.517	0.052	13.83	0.483		
	0.722	0.521	0.052	13.88	0.479		
PEB						0.649	0.953
	0.815	0.664	0.039	20.90	0.336		
	0.783	0.613	0.041	19.10	0.387		
	0.869	0.755	0.032	27.16	0.245		
	0.794	0.630	0.038	20.89	0.370		
	0.736	0.542	0.054	13.63	0.458		
	0.842	0.709	0.035	24.06	0.291		
	0.828	0.686	0.039	21.23	0.314		
	0.863	0.745	0.034	25.38	0.255		
	0.846	0.716	0.037	22.86	0.284		
	0.726	0.527	0.051	14.24	0.473		
	0.747	0.558	0.046	16.24	0.442		
A.V						0.634	0.933
	0.754	0.569	0.042	17.95	0.431		
	0.782	0.612	0.038	20.58	0.388		
	0.741	0.549	0.044	16.84	0.451		
	0.736	0.542	0.045	16.36	0.458		
	0.828	0.686	0.037	22.38	0.314		
	0.846	0.716	0.035	24.17	0.284		
	0.833	0.694	0.036	23.14	0.306		
	0.842	0.709	0.034	24.76	0.291		
ESTL						0.582	0.939
	0.739	0.546	0.037	19.97	0.454		
	0.727	0.529	0.046	15.80	0.471		
	0.817	0.667	0.041	19.93	0.333		
	0.719	0.517	0.052	13.83	0.483		
	0.734	0.539	0.039	18.82	0.461		
	0.749	0.561	0.038	19.71	0.439		
	0.829	0.687	0.033	25.12	0.313		
	0.733	0.537	0.038	19.29	0.463		
	0.815	0.664	0.051	15.98	0.336		
	0.762	0.581	0.040	19.05	0.419		
	0.756	0.572	0.039	19.38	0.428		

Notes: λ = Item loadings, C.R = composite reliability, ∑λ^2^ = sum of square of item loadings, E-Variance = error variance, CSR = corporate social responsibility, PEB = pro-environmental behavior, ESTL = environmental specific transformational leadership, A.V = altruistic values.

**Table 2:** Correlations and discriminant validity.

Construct	CSR	PEB	A.B	ESTL	Mean	SD
CSR	0.762	0.398 **	0.377 **	0.396 **	4.97	0.62
PEB		0.806	0.528 **	0.482 **	5.08	0.67
A.V			0.796	0.336 **	4.49	0.74
ESTL				0.776 **	5.19	0.59

Notes: SD = standard deviation, ** = significant values of correlation, bold diagonal = discriminant validity values.

**Table 3:** Model fit comparison, alternate vs. hypothesized models.

Model	*χ* ^2^	*Df*	*χ*^2^/*df*	Δ*χ*^2^*/df*	NFI	CFI	RMSEA
4-factor	1709.355	898	1.903	_	0.957	0.959	0.039
3-factor	1949.238	722	2.699	0.796	0.882	0.891	0.042
2-factor	2424.592	645	3.759	1.060	0.827	0.819	0.0722
1-factor	2704.619	510	5.303	1.544	0.653	0.678	0.089

**Table 4:** Direct effect structural model results.

Hypotheses	Relationship Nature	Beta-Value (SE)	CR	*p*-Value	CI	Decision
H1: CSR→PEB	+	(*β*1) 0.411 ** (0.039)	10.538	***	0.428–0.497	Accepted
H2: CSR→ESTL	+	(*β*2) 0.407 ** (0.038)	10.710	***	0.395–0.428	Accepted
H3: ESTL→PEB	+	(*β*3) 0.476 ** (0.034)	14.000	***	0.287–0.299	Accepted

Notes: CI = 95% confidence interval with lower and upper limits, ** = significant beta values *** = significant *p*-values.

**Table 5:** Mediation and conditional effects.

Path	Estimates	S.E	Z-Score	*p*-Value	CI	Decision
H4: CSR→ESTL→PEB	(*β*4) 0.194 **	0.023	8.423	***	0.211–0.266	Accepted
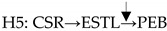	(*β*5) 0.268 **	0.020	13.40	***	0.239–0.308	Accepted

Notes: CI = 95% confidence interval with lower and upper limits, ** = significant beta values, *** = significant *p*-values S.E = standard error.

## Data Availability

The data can be made available on a reasonable request by contacting the corresponding author.
